# Agreement Between Bioelectrical Impedance Analysis and Ultrasound for Measuring Body Composition in Women with Breast Cancer

**DOI:** 10.3390/diagnostics15121545

**Published:** 2025-06-17

**Authors:** Jared Rosenberg, Jyotsna Natarajan, David J. Carpenter, Chris Peluso, Christie Hilton, Colin E. Champ

**Affiliations:** 1 Kinesiology Department, SUNY Cortland, Cortland, NY 13045, USA; 2 Exercise Oncology Consortium, Pittsburgh, PA 15232, USA; davidcarpenter522@gmail.com (D.J.C.); christopher.peluso@ahn.org (C.P.); 3 Drexel University College of Medicine, Philadelphia, PA 19129, USA; jmn353@drexel.edu; 4 Department of Radiation Oncology, Wellstar Paulding Medical Center, Hiram, GA 30141, USA; 5 Allegheny Health Network Cancer Institute, Exercise Oncology and Resiliency Center, Pittsburgh, PA 15202, USA; 6 Department of Medical Oncology, Allegheny Health Network, Pittsburgh, PA 15212, USA; christie.hilton@ahn.org; 7 Department of Radiation Oncology, Allegheny Health Network, Pittsburgh, PA 15212, USA

**Keywords:** ultrasound, bioelectrical impedance analysis, cancer, body composition

## Abstract

**Background/Objectives:** Higher percent body fat (BF) is associated with worse outcomes after treatment for breast cancer (BC). While ultrasound (US) imaging is a reliable method for analyzing body composition, it requires trained individuals for utilization. As such, bioelectrical impedance analysis (BIA) has been suggested as an alternative. Therefore, the goal of this study was to compare BIA with US. **Methods**: Women from three prospective exercise BC studies were analyzed with US and BIA before an exercise intervention. Spearman’s correlation was used as a nonparametric measure to examine bivariate relationships between percent body fat measured by BIA and US. **Results**: In total, 106 women with BC had their body composition measured using both US and BIA. Despite a strong correlation between the two methods (r = 0.8, *p* < 0.01), US reported lower mean percent BF vs. BIA (34.6 ± 0.7% vs. 38.0 ± 0.8%, *p* < 0.01). In a subgroup analysis, concordance was seen in women with a body mass index below (BMI) ≤ 26 kg/m^2^. BIA overreported percent BF compared to US in women with a BMI > 26 kg/m^2^. **Conclusions**: In women with BC and BMI ≤ 26, US and BIA are in concordance when measuring BF. In women with a BMI > 26, BIA reports a higher BF than US. Overall, there was a strong correlation between modalities, while BMI can be used to guide the utilization of BIA as an alternative to US for assessing body composition.

## 1. Introduction

Obesity is associated with worse outcomes after the treatment for breast cancer [1,2]. Body composition, and specifically lower adipose tissue, is associated with lower mortality after breast cancer treatment [3]. These findings highlight the importance of accurately tracking body composition during breast cancer treatment. Ultrasound (US) imaging is a common method of collecting body composition that has been shown to be a reliable and valid measure that can be used to quantify muscle and fat tissue compared to MRI [4]. Exercise interventions in individuals with cancer have utilized US to track changes in body composition [5,6,7,8]. However, US is not widely available and requires adequate training [9].

Another common measure used to assess body composition that has been used in the cancer setting is bioelectrical impedance analysis (BIA). Although BIA is more expensive, it is more readily available and does not require specially trained staff to administer the assessment [10]. While BIA has been used for assessing body composition in the cancer setting, its use in individuals with breast cancer presents two unique challenges [11]. The first challenge is anthropometric, as individuals with breast cancer commonly have higher body mass indices (BMI). In individuals with higher BMIs, BIA has been shown to underestimate percent body fat compared to dual X-ray absorptiometry (DXA) [12,13,14]. The second challenge is treatment-related lymphedema, characterized by excessive fluid retention in the upper extremity due to dysregulated lymphatic flow. Lymphedema is estimated to affect one out of every five women treated for breast cancer [15,16,17]. BIA measures the impedance of a low-level electrical current that passes through the body. Fat tissue, with a lower water content than muscle tissue, shows more resistance. Consequently, individuals with more muscle than fat tissue have lower resistance, and therefore a lower body fat percentage [10]. Thus, it could be assumed that BIA measurements might overestimate lean body mass in the presence of lymphedema, leading to underestimations of the percent of body fat.

Therefore, the aims of this study were to (1) compare BIA and US measurements of body fat percentage in individuals with breast cancer, and (2) assess the degree to which lymphedema and BMI affect body fat percentage measurements across these modalities.

## 2. Materials and Methods

### 2.1. Participants

Women aged 20–89 with biopsy-proven ductal carcinoma in situ (DCIS) or invasive breast cancer were eligible for this cross-sectional analysis. All women were enrolled in one of three prospective exercise trials. The data used for this analysis were collected after enrollment in the study, but prior to the initiation of the exercise intervention. Participants actively undergoing radiation therapy, anti-estrogen, and targeted systemic therapy were allowed to participate, while participants actively receiving systemic cytotoxic chemotherapy were excluded. All participants were screened by study personnel during oncologic consultation or a subsequent follow-up visit. The full details of the participants have been described previously [18].

Recruitment took place between 15 September 2022 and 16 July 2024, at the Allegheny Health Network (AHN) departments of surgical, medical, and radiation oncology, along with the AHN Cancer Institute Exercise Oncology and Resiliency Center (EOC). Consent was obtained for each participant before enrollment in the study. All studies were institutional review board-approved and registered at ClinicalTrials.gov (NCT05747209 [Approved 28 December 2022], NCT05978960 [Approved 20 May 2023], and NCT06083324 [Approved 2 March 2023]).

#### Body Composition Analysis

All participants underwent body composition analysis via an InBody 970 BIA machine (InBody Co., Seoul, Republic of Korea), using 3 MHz frequency. Body composition analysis included total body fat (lbs) and total muscle mass (lbs). Additionally, percent body fat was measured via US utilizing Body Metrix software V.5.7.11043 (IntelaMetrix Inc., Brentwood, CA, USA) [19]. Measurements for the US were collected at the triceps, suprailiac, abdominal, and thigh utilizing the Jackson & Pollack Calculations [20], which measure only subcutaneous adipose tissue (SAT), and not visceral adipose tissue (VAT). Body fat collected at these sites via an US, utilizing Body Metrix software, has demonstrated strong reproducibility, and has been validated against body fat collected via DXA [21,22].

When assessing percent body fat collected via BIA, individuals were categorized by both lymphedema status (non-lymphedema vs. lymphedema) and obesity status (non-obese vs. obese). Clinical lymphedema was defined as a 3% increase in arm measurement and was measured routinely at our lymphedema centers. This conservative threshold triggers the aggressive management of lymphedema, including massage and sleeve usage.

### 2.2. Statistical Analysis

Spearman’s correlation was used as a nonparametric measure to examine bivariate relationships between percent body fat measured by BIA and US. The correlation analysis was followed by an application of Fisher’s z-transformation of the correlation coefficients to determine significant differences in correlation between obesity and lymphedema status. Since BIA and US are not gold standard measures of assessing body composition, a Demin regression was performed to assess the agreement of both measures. A dependent *t*-test was used to assess the mean difference between body fat measured by BIA compared to US. Next, due to the suggestion that BIA is less accurate in women with obesity, and the potential for hydration status to influence BIA accuracy, we used a two-way analysis of variance (ANOVA) to examine the effect of obesity and lymphedema status on percent body fat collected via BIA [10,14]. This allowed us to assess whether there is a differential effect of obesity and/or lymphedema status on percent body fat collected via BIA. Data that did not meet the assumptions for normality were log10 transformed. Data were analyzed using SPSS 29.0 and RStudio version 4.4.1 for the analysis of descriptive statistics, comparison of means, correlations, and ANOVAs, with significance set at *p* ≤ 0.05.

### 2.3. Power Size Calculation

The sample size was estimated using data from the BC cohort (*n* = 44), with body fat percentage as the main variable. The mean and standard deviation of body fat percentage measured via BIA and US were 37.6 ± 9.0 and 35.8 ± 6.9, respectively, with a correlation of 0.78. These values yielded an effect size of 0.319. Using an α level set at 0.05 and a desired power of 0.95, the power analysis indicated a required sample size of 108, corresponding to an actual power of 0.95, and a critical t value equal to 1.65.

## 3. Results

In total, 106 participants were analyzed for the study, with descriptive statistics listed in Table 1. The mean patient age was 55.5 years (range, 24–79 years), with a mean BMI of 29.4 kg/m^2^. Of 105 (24%) participants, 25 were found to have upper-extremity lymphedema at baseline. No women experienced significant clinical lymphedema beyond stage 1.

**Figure 1:**
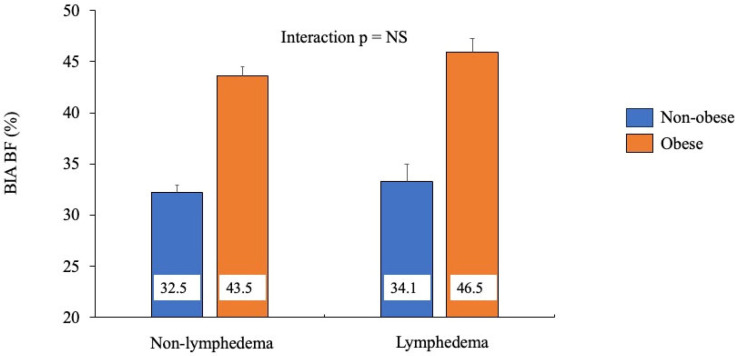
Comparing percent body fat by lymphedema phenotype, measured by bioelectrical impedance, across obesity status.

To assess for potential interaction, BIA percent body fat was assessed with respect to lymphedema and (non-)obese status via 2-way ANOVA (Figure 1). Amongst individuals without lymphedema (*n* = 81), those without obesity had 25.3% less body fat compared to individuals with obesity. Amongst individuals with lymphedema (*n* = 25), those without obesity had 26.7% less body fat compared to individuals with obesity. Therefore, no interaction effect was noted (Figure 1). Furthermore, a small effect size was noted, η^2^ = 0.00027.

Next, within each lymphedema status, individuals were further grouped based on their BMI category (normal weight, overweight, obese). Among individuals without lymphedema, there was a steady decrease in percent of body fat from individuals with obesity to those with overweight and those with normal weight (43.6, 36.0, and 30.2%, respectively). A similar decrease in percent body fat was seen in individuals with lymphedema (45.9, 36.0, and 30.9%, respectively). Therefore, a significant interaction was not observed.

**Figure 2:**
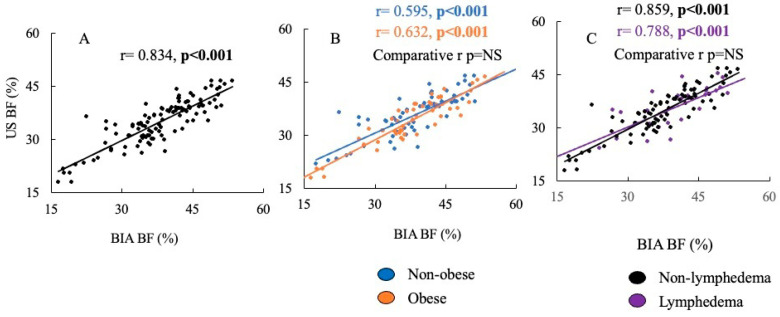
The correlation of percent body fat measured by ultrasound vs. BIA. Graph (**A**) is the whole cohort. Graph (**B**) compares the correlations of percent body fat measured by ultrasound vs. BIA in individuals without obesity compared to individuals with obesity. Graph (**C**) is the percent of body fat measured by ultrasound vs. BIA in individuals without lymphedema compared to individuals with lymphedema.

Spearman’s correlation revealed a strong positive relationship between percent body fat across BIA and US (*p* < 0.001) (Figure 2, graph A). When comparing the Spearman correlations between percent body fat measured by BIA versus US in individuals with and without obesity, no difference was found between the strength of the correlations (Figure 2, graph B). Similarly, no difference was found between the strength of the correlations between percent body fat measured by BIA and US in individuals with and without lymphedema (Figure 2, graph C). Deming regression analysis revealed no fixed bias, as the intercept was 3.71 [−1.23, 8.65] (95% CI). However, proportional bias was present as the slope was 0.815 [0.688, 0.942], suggesting there was no strong agreement between the two methods.

**Figure 3:**
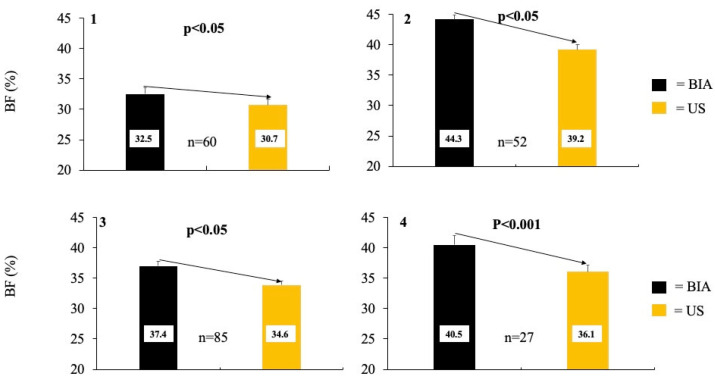
The averages of percent body fat collected via Bioelectrical impedance (BIA) compared to ultrasound (US). Graph (**1**) compares percent body fat collected via BIA vs. US in individuals without obesity, while graph (**2**) concerns individuals with obesity. Graph (**3**) compares percent body fat collected via BIA vs. US in individuals without lymphedema, while graph (**4**) is in individuals with lymphedema.

When comparing percent body fat measured by BIA versus US, BIA overestimated body fat percentage compared to US across all participants (38.0 ± 0.8% vs. 34.6 ± 0.7%, *p* < 0.001) (Table 1). When comparing percent body fat measured by BIA versus US by obesity status (non-obese vs. obese), BIA measurements were significantly higher for both phenotypes (32.5 ± 0.9% vs. 30.7 ± 0.7% and 44.3 ± 0.8% vs. 39.2 ± 0.9%, respectively, both *p* < 0.001). Similarly, when comparing percent body fat measured by BIA versus US across lymphedema status (non-lymphedema vs. lymphedema), BIA was significantly higher for both phenotypes compared to US (37.1 ± 0.9 vs. 33.9 ± 0.8% and 40.8 ± 1.6 vs. 37.0 ± 1.1%, respectively, both *p* < 0.001) (Figure 3). Furthermore, when comparing percent body fat measured by BIA versus US by obesity and lymphedema status, BIA reported higher body fat percentages for all phenotypes (*p* < 0.01), except for non-obese individuals with lymphedema (33.3 ± 2.0% vs. 32.4 ± 1.7%). Lastly, when comparing percent body fat measured by BIA versus US across BMI categories (normal weight vs. overweight vs. obese), BIA reported higher body percentages in all weight categories (*p* < 0.05), except for individuals with normal weight (30.2 ± 0.9 vs. 30.0 ± 0.8%).

## 4. Discussion

The current analysis of women with breast cancer revealed a strong correlation between measurements of percent body fat collected via BIA and US, regardless of obesity or lymphedema status. Overall, BIA consistently showed higher percent body fat compared to US, regardless of obesity or lymphedema status.

The present data do not support the use of BIA in lieu of US for measuring body fat percentages among women with breast cancer. As in the whole cohort, BIA consistently overestimated percent body fat compared to US. Furthermore, as suggested by the Deming regression, for every one unit increase in the BIA, a 0.815 increase was observed for US. Even when individuals were categorized by obesity (obese vs. non-obese) and lymphedema status (lymphedema vs. non-lymphedema), a higher percent body fat was consistently found with BIA (Figure 3). No interaction effect was observed across obesity or lymphedema status on percent body fat measured via BIA.

Our findings differ from those of Bondareva et al. [23], who observed a similar percent body fat when collected via BIA vs. US in 206 women without cancer aged 18–67 years. This discrepancy may be a result of a different method of BIA collection using a different frequency. Bondareva et al. [23] used “wrist–ankle” electrodes at 50 kHZ frequency, placed on the right side of the body while subjects were supine [24]. Higher frequencies can penetrate human cells more effectively than lower frequencies, making them more accurate for measuring intracellular water results [10]. Another possible explanation for the different findings between our study and in Bondareva et al. [23] may be the difference in mean body fat percentage among participants in the two studies. The mean percent body fat in participants in the Bondareva et al. [23] study was ~31%, while the current study’s mean percent body fat was ~36%. Our data suggest that BIA yields a higher percent body fat in individuals with elevated percent body fat (i.e., above 30%) compared to US. It should be noted that in the current study, the mean BMI for women with a percent body fat less than 30% was 22.5 kg/m^2^, which is similar to the mean BMI (~26 kg/m^2^) of the total cohort in the study by Bondareva et al. [23]. These findings suggest that for women with breast cancer who have a BMI ≤ 26 kg/m^2^, percent body fat can be measured with similar accuracy using either BIA or US. Supporting this suggestion is the finding that when the sample was restricted to only women with a BMI < 26 kg/m^2^ (*n* = 34), no difference was found between BIA and US regarding the assessment of body fat percentage.

The strengths of this current cross-sectional investigation are as follows: (1) it is a first-time assessment comparing the agreement of BIA and US in measuring percent body fat in women with breast cancer, (2) it is a first-time assessment comparing measurements of percent body fat collected via BIA in women with lymphedema vs. without lymphedema, and (3) it comprises a large cohort with a wide range of ages and adiposity with varying stages of breast cancer. One potential limitation could be that the Jackson and Pollack method for estimating percent body fat has been shown to undervalue the actual percent body fat compared to measurements obtained by DXA [24,25,26]. In the current study, we assessed percent body fat via a US with collection at the Jackson and Pollack anatomical sites, which measures SAT [27]. One of the major physiological roles of SAT is the storage of excess triglycerides. When SAT cannot expand to accommodate an excess amount of energy, VAT and ectopic deposits occur [28]. In women aged 18–64, BMI has been shown to be significantly correlated with total VAT amount, suggesting that women with higher BMIs have more VAT than women with lower BMIs [29]. Therefore, percent body fat measured via US potentially did not capture the total VAT amount due to the employment of subcutaneous collection sites, potentially leading to an under-reported percentage of body fat in women with higher BMIs. Such under-reporting may not have occurred in measurements obtained via BIA due to the whole-body approach, as opposed to the site-specific measurements of US. The higher VAT amount in individuals with higher BMIs, compared to those with lower BMIs, alongside the subcutaneous collection sites of US potentially explain why percent body fat measurements from BIA and US were similar in women with a BMI under 26 kg/m^2,^ but differed in women with BMIs greater than 26 kg/m^2^. Another limitation is that both US and BIA are considered two-compartment (2C) models, which divide the body into fat mass and fat-free mass. Accordingly, both BIA and US are not direct measurements of body fat. BIA estimates body fat from a calculation regressed from total body water to fat-free mass to fat mass, and then to body fat percent, while US uses subcutaneous fat at specific sites to estimate whole-body fatness and body fat percentage [9,10]. In contrast, 4C models divide body weight into fat, water, mineral and protein, and are more robust to interindividual variabilities in the composition of fat free mass and thus considered more reliable. However, US and BIA are 2C models, but both have been validated against 4C models, suggesting both measures are accurate ways to assess body composition [30,31].

Our findings suggest two important clinical implications for an individual with breast cancer and a BMI > 26 kg/m^2^. First, if BIA is used to assess body composition in obese individuals, a higher value will be measured than if collected via US. Secondly, due to the lack of agreement, the same measurement tool (either BIA or US) should be used consistently when assessing changes in body composition over time, and one should consider utilizing both methods to account for discrepancies, which is the standard of care at our exercise facility [32].

## 5. Conclusions

Our cross-sectional analysis of women with breast cancer revealed that BIA measurements yielded higher percent body fat compared to US. No interaction effect was observed for lymphedema and obesity status with respect to body fat percentage collected via BIA. Subgroup analysis revealed that in women with a BMI ≤ 26, the percent body fat values measured via BIA and US were similar, while for women with a BMI > 26, BIA yielded a higher percent body fat versus US, suggesting the importance of using the same mechanisms for measurement and potentially utilizing multiple measurement methods.

## Figures and Tables

**Table 1:** Descriptive characteristics.

Physical Characteristics	*N*	Mean	Minimum	Maximum
Age (years)	106	55.5 ± 1.1	24	79
Lymphedema (Y/N)	25/81			
Obese/Overweight/Normal Weight	49/23/34			
BMI (kg/m^2^)	106	29.4 ± 0.6	17.3	48.1
BIA body fat (%) *	106	38.0 ± 0.8	16.6	53.5
US body fat (%)	106	34.6 ± 0.7	17.4	49.2

BIA = bioimpedance analysis, BMI = body mass index, US = ultrasound * Significantly higher than US.

## Data Availability

The data that support the findings of this study are available from the corresponding author upon reasonable request.

## Abbreviations

The following abbreviations are used in this manuscript:

BF	Body fat
US	Ultrasound
BIA	Bioelectrical impedance analysis
BMI	Body mass index
